# A *MANBA *mutation resulting in residual beta-mannosidase activity associated with severe leukoencephalopathy: a possible pseudodeficiency variant

**DOI:** 10.1186/1471-2350-10-84

**Published:** 2009-09-03

**Authors:** Frédérique Sabourdy, Pierre Labauge, Hilde Monica Frostad Riise Stensland, Michèle Nieto, Violeta Latorre Garcés, Dimitri Renard, Giovanni Castelnovo, Nicolas de Champfleur, Thierry Levade

**Affiliations:** 1 Laboratoire de Biochimie 'Maladies Métaboliques', Institut Fédératif de Biologie, CHU Purpan, Toulouse, France; 2 INSERM U858, Institut de Médecine Moléculaire de Rangueil, Université Toulouse III Paul-Sabatier, IFR31, Toulouse, France; 3 Service de Neurologie CHU de Nîmes, Nîmes, France; 4 Department of Medical Genetics, University Hospital of North-Norway, Tromsø, Norway

## Abstract

**Background:**

β-Mannosidosis (OMIM 248510) is a rare inborn lysosomal storage disorder caused by the deficient activity of β-mannosidase, an enzyme encoded by a single gene (*MANBA*) located on chromosome 4q22-25. To date, only 20 cases of this autosomal recessive disorder have been described and 14 different *MANBA *mutations were incriminated in the disease. These are all null mutations or missense mutations that abolish β-mannosidase activity. In this study, we characterized the molecular defect of a new case of β-mannosidosis, presenting with a severe neurological disorder.

**Methods:**

Genomic DNA was isolated from peripheral blood leukocytes of the patient to allow *MANBA *sequencing. The identified mutation was engineered by site-directed mutagenesis and the mutant protein was expressed through transient transfection in HEK293T cells. The β-mannosidase expression and activity were respectively assessed by Western blot and fluorometric assay in both leukocytes and HEK293T cells.

**Results:**

A missense disease-associated mutation, c.1922G>A (p.Arg641His), was identified for which the patient was homozygous. In contrast to previously described missense mutations, this substitution does not totally abrogate the enzyme activity but led to a residual activity of about 7% in the patient's leukocytes, 11% in lymphoblasts and 14% in plasma. Expression studies in transfected cells also resulted in 7% residual activity.

**Conclusion:**

Correlations between MANBA mutations, residual activity of β-mannosidase and the severity of the ensuing neurological disorder are discussed. Whether the c.1922G>A mutation is responsible for a yet undescribed pseudodeficiency of β-mannosidase is also discussed.

## Background

β-Mannosidosis (OMIM 248510) is a rare autosomal recessive disease due to the deficient activity of acid β-mannosidase, a lysosomal hydrolase involved in the terminal catabolism of glycoproteins [1-3]. It is characterized by intralysosomal accumulation of (di)saccharides. Despite the low number of human cases reported so far (21 cases in 17 families including this study), a wide range of symptoms of varying degree of severity is observed [2,4-19]. The disease was first described in ruminants [1,20,21], where the enzyme defect is associated with a severe neurological phenotype including demyelination, skeletal deformation, facial dysmorphism and neonatal death. The human disease is generally of milder severity and with a later onset. Whereas mental retardation, behavioural abnormalities and hearing loss are frequently observed, facial dysmorphism, skeletal deformation, susceptibility to respiratory infections and skin lesions have only been reported in a few patients.

In this study, we analyzed the molecular defect underlying a new case of deficient activity of β-mannosidase with marked involvement of pyramidal and cerebellar pathways, a clinical spectrum that has not previously been reported in β-mannosidosis. A missense mutation in the *MANBA *gene was identified. This is the first report of a *MANBA *missense mutation present at the homozygous state and which retains a residual activity while being associated with a severe neurological disease.

## Methods

### Case report

The clinical presentation of the case has been reported elsewhere [22]. Briefly, the patient was born to Algerian consanguineous (first cousins) parents. Mental retardation was observed since the age of 4. Spastic tetraparesis and cerebellar ataxia were noted at age 12, along with visual and hearing deficits. Symptoms progressively worsened and, at age 26, the patient suffered from tetraplegia, dysphagia and dysarthria. Brain MRI showed a striking cortical and subcortical atrophy while there was no noticeable demyelination. Skin lesions and skeletal deformations were absent. Siblings were asymptomatic. Written informed consent was obtained from the patient's father for the studies described below and for publication of the study, according to the protocols approved by the ethics review board of the Toulouse Hospital. Research was performed in compliance with the Declaration of Helsinki.

### Enzyme assays

β-Mannosidase activity in peripheral blood leukocytes, cultured lymphoid cells, plasma and HEK293T cells was assessed using 4-methylumbelliferyl-β-D-mannopyranoside (Sigma, St Quentin Fallavier, France) as previously described [12]. Acid β-galactosidase and *E. coli *β-galactosidase activities were determined using the 4-methylumbelliferyl-β-D-galactopyranoside substrate (Sigma). Briefly, cell lysates were incubated in the presence of 0.4 mg/mL of substrate at acidic (0.1 M sodium acetate buffer, pH 4.5) or neutral (0.1 M Tris/HCl buffer, pH 7.0) pH for determining acid, lysosomal β-galactosidase and *E. coli *β-galactosidase activities, respectively. For the bacterial enzyme (the activity of which is cation-dependent), assays were performed either in the presence of 4 mM EDTA or 10 mM MgCl_2_, and the activity was calculated by subtracting the value obtained in the presence of the chelator from that obtained in the presence of MgCl_2_. Protein concentration was determined using the bicinchoninic acid protein assay (Sigma).

### Molecular analyses

Genomic DNA was isolated from peripheral blood leukocytes (Nucleospin II, Macherey-Nagel, Germany). Exons 1 through 17 of the *MANBA *gene including intron-exon junctions were individually amplified and sequenced in both directions using previously described primers [23]. For cDNA analysis, RNA was isolated from Epstein-Barr virus-transformed lymphoid cells (SV Total RNA extraction kit, Promega), reverse-transcribed and amplified using previously reported primers [23]. DNA sequencing was performed using an ABI3100 Applied Biosystems automatic sequencer. For restriction enzyme analysis of exon 14, amplicons were incubated with *MaeIII *or *BbvI *(Roche Diagnostics, Germany, and New England Biolabs, MA, respectively), and analysed on a 1.8% agarose gel. Exon 14 from 200 control subjects, corresponding to 61 Finnish, 57 Norwegian, 35 North-African, 18 Hondurian, 15 Palestinian, and 14 Polish control individuals (anonymized blood-donors), was PCR-amplified and sequenced in the forward direction as previously described [23]. The programs SIFT  and PolyPhen  were used for evaluation of the p.Arg641His substitution.

### Site-directed mutagenesis of the human *MANBA *cDNA

The pCS2-MANBA-WT plasmid was kindly provided by Dr. T. Beccari [24]. This construct was used as template for site-directed mutagenesis using the QuickChange Site-Directed Mutagenesis Kit (Stratagene) and primer pairs (Forward primer 5' CTACCGCCGTAGTCACAGCGAGATAGTGG 3' and Reverse primer 5' CCACTATCTCGCTGTGACTACGGCGGTAG 3') to introduce the c.1922G>A mutation and to create the pCS2-MANBA-p.Arg641His expression construct. The recombinant vector was purified (Qiagen Plamid Maxi kit) and the insert sequenced using the following primers: 5' GCAAATGGGCGTTCCATTG 3', 5' ACTGCCCTCCACTTGTGC 3', 5' GTGGATGCTAATATGAATAC 3', 5' CATCACGAAGGTGGTAAC 3', and 5' GACCAACTACCACTTCTTG 3'.

### Cell lines and transfections

Human embryonic kidney cells (HEK293T) were grown in a humidified 5% CO_2 _atmosphere at 37°C in DMEM containing Glutamax (2 mM), penicillin (100 U/ml), streptomycin (100 μg/ml) and 10% heat-inactivated FCS. Cells were transfected using Superfect (Qiagen) with 1 μg of pCMV-LacZ plasmid and 5 μg of either pCS2-MANBA or pCS2-MANBA-p.Arg641His plasmid. After 48 hours incubation, cells were washed with PBS, harvested and cell pellets were frozen at -80°C until use. Cells were lysed in water, and enzyme activities were determined as described above. For Western-blot analyses, cells were lysed in lysis buffer (Cell Signaling) containing 1 mM PMSF and a protease inhibitor cocktail (Complete, Roche).

### Western Blot analyses

Cell lysates were separated using a 7.5% SDS-polyacrylamide gel and transferred to a nitrocellulose membrane (Bio-Rad). Proteins were detected using an ECL detection system (Pierce). β-Mannosidase was detected by using a mouse polyclonal antibody (1:1000, Abnova). An anti-β-actin (1:1000, Sigma) was used as a control for protein loading. Goat anti-rabbit and anti-mouse secondary antibodies (1:3000) were from Cell Signaling.

## Results

### Enzyme studies

The β-mannosidase enzymatic defect was present both in the patient's cells (i.e., peripheral blood leukocytes and Epstein-Barr virus-transformed lymphoid cells) and plasma (Table 1). Of note, a residual activity of about 7 to 14% was consistently observed in all samples.

**Table 1:** β-Mannosidase activity in the family.

**Patient**	**β-Mannosidase activity**		
	**Leukocytes**	**Lymphoid cells**	**Plasma**

I-1 (father)	51; 42	20	115; 90
I-2 (mother)	50; 35	16	75; 51
II-1	51; 60	29	127; 102
II-2	4; 8	6; 7	24
II-3	92; 167	ND	160; 115
II-4	68	ND	93; 84
Controls	86 ± 26 (n = 25)	58 ± 19 (n = 4)	174 ± 45 (n = 12)

**Figure 1:**
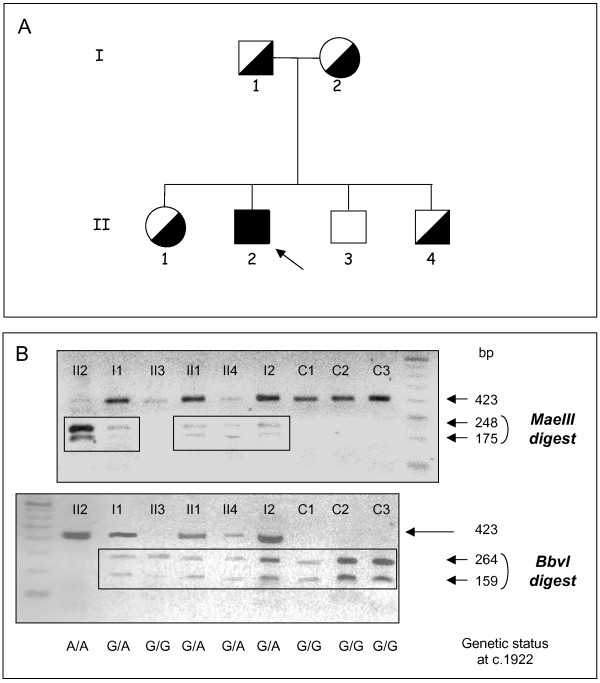
**Segregation of the c.1922G>A *MANBA *mutation in the family**. A, Family pedigree. The arrow denotes the proband. Genetic status for the c.1922G>A mutation is indicated by black and white symbols. B, Restriction enzyme analysis of exon 14 in the patient, his family and three control subjects (denoted C1 to C3). PCR-amplified exon 14 (a 423 bp fragment) was digested with *MaeIII *or *BbvI *and analyzed by gel electrophoresis.

β-Mannosidase activity was determined in peripheral blood leukocytes, Epstein-Barr virus-transformed lymphoid cells and plasma from family members (see Figure 1A). Enzyme activity is expressed as nmol/h.mg of protein (for cells) or nmol/h.ml (for plasma). For patients, the different values correspond to independent experiments; for controls, means ± SEM of the indicated number of different samples. ND, not determined.

These results led us to investigate the β-mannosidase activity in the asymptomatic family members (see pedigree in Figure 1A). Intermediate levels of β-mannosidase activity were found in the peripheral blood leukocytes of both parents, as well as in the youngest brother (II-4) and sister. The remaining brother (II-3) had rather normal β-mannosidase activity (Table 1). Determination of β-mannosidase activity in plasma and Epstein-Barr virus-transformed lymphoid cells of each relative gave concording results.

### Mutational analysis

As we recently reported [22], sequencing of all exons, the corresponding exon-intron junctions and the flanking regions of the *MANBA *gene showed that the proband was homozygous for the novel mutation c.1922G>A in exon 14. This mutation, which was also found by sequencing the cDNA obtained from the patient's cultured lymphoid cells, caused substitution of arginine 641 by histidine (p.Arg641His). The proband's parents, his sister and his youngest brother (II-4) were all heterozygous for this mutation, in agreement with the intermediate level of β-mannosidase activity observed in their cells or plasma. The remaining brother (II-3), who exhibited a normal β-mannosidase activity level, did not carry the c.1922G>A mutation. The genetic status of the family members was confirmed by restriction enzyme analysis using the *BbvI *and *MaeIII *enzymes which cleave the wt and mutant sequence, respectively (Figure 1B). The c.1922G>A mutation was not present among 400 control alleles, and two www-based prediction programs suggested that the p.Arg641His change was pathogenic.

The patient was also homozygous for the sequence variant c.2102C>T (p.Thr701Met) in exon 15 (not shown). This sequence variant is common in the control population (frequency 0.49) and is not associated with beta-mannosidosis [24].

### Expression studies

In order to test whether the c.1922G>A mutation could be responsible for the β-mannosidase deficiency in the proband, the wt and mutant enzymes were transiently expressed in HEK293T cells and the resulting enzyme activity was measured. As a control for transfection efficiency, cells were simultaneously transfected with a vector encoding the bacterial β-galactosidase (lacZ). The endogenous, acid lysosomal β-galactosidase activity was also assessed as a reference for enzyme activity.

**Figure 2:**
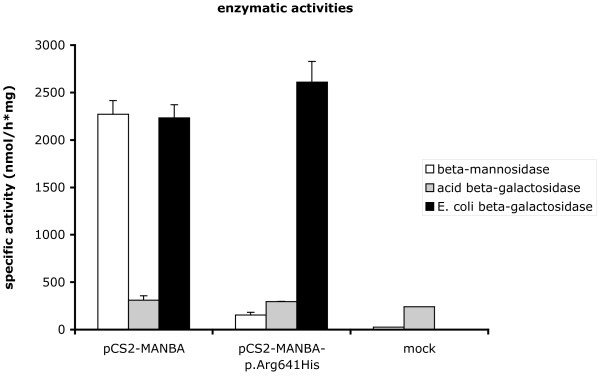
**Enzymatic activities in transfected HEK293T cells**. A, Acid β-mannosidase, acid β-galactosidase and *E. coli *β-galactosidase activities were measured in lysates of untransfected HEK293T cells and cells over-expressing either the wild-type (pCS2-MANBA) or the mutant (pCS2-MANBA-p.Arg641His) acid β-mannosidase. Activities are expressed as nmol/h.mg protein, and are means ± SEM of 3 independent experiments performed in duplicate. The β-mannosidase activity was similar in untransfected cells and cells transfected only with the vector encoding the bacterial β-galactosidase (data not shown).

As illustrated in Figure 2, whereas expression of the wt cDNA sequence led to a considerable increase (about 80-fold) of β-mannosidase activity, transfection of the vector carrying the c.1922G>A mutation resulted in a much lower activity, indicating that this mutation is possibly disease-causing. However, the enzyme activity generated by the mutated cDNA remained significantly higher (about 5.6-fold) than that in untransfected cells. The acid β-galactosidase activity was similar in all the cell samples analysed.

**Figure 3:**
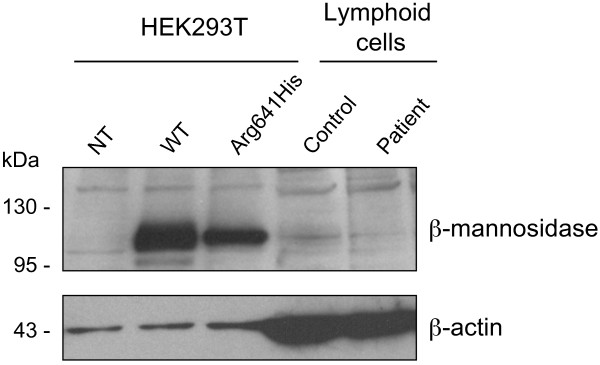
**Expression of endogenous and overexpressed β-mannosidase**. Lysates of control and patient's lymphoid cells (100 μg protein), as well as HEK293T cells transiently transfected with a vector encoding wild-type (pCS2-MANBA) or mutant (pCS2-MANBA-p.Arg641His) β-mannosidase (25 μg protein), were assessed for β-mannosidase expression by Western-blot analysis. NT, not transfected.

The mutant β-mannosidase expression was also characterized by Western-blotting, both in the patient's lymphoid cells and in transiently transfected HEK293T cells (Figure 3). Both the overexpressed and endogenous mutant protein was detected, showing the same apparent size (about 101 kDa) as the wt enzyme. In HEK293T cells, however, the mutant β-mannosidase was less abundant than the wt, suggesting a possible instability of the mutant protein. A similar conclusion could not be drawn for the endogenous enzyme since its expression in lymphoid cells was very low.

## Discussion

Since the identification of the *MANBA *gene on chromosome 4q21-q25, which encodes for the human lysosomal β-mannosidase, a number of *MANBA *mutations have been described, and attempts to establish genotype-phenotype correlations have been proposed [23,25] (see additional file 1: table for enzyme activity and clinical phenotype associated by MANBA mutations.). Among the 14 β-mannosidosis-associated sequence variants identified so far, most are null mutations [9,13,16,18,23-25]. Only three disease-causing missense mutations have been reported to date, i.e., p.Arg182Trp [17], p.Gly392Glu [9] and p.Ser505Pro [24], but all three were associated with null mutations in compound heterozygous patients (see additional file 1). In this study, we identified a missense *MANBA *mutation, which was associated with an uncommon phenotype. This is the first report of a missense mutation causing deficient activity of β-mannosidase in a homozygous context, and interestingly, it is also the first report of a missense mutation resulting in a residual *in vitro *enzyme activity of β-mannosidase.

The first hypothesis that proposed *MANBA *null mutations could be responsible for the most severe phenotypes was ruled out when such defects were also shown to be associated with mild symptoms [9]. It is noteworthy however, that all the disease-causing mutations identified to date, totally or almost totally abrogate β-mannosidase activity (see additional file 1). These observations may erroneously lead to the conclusion that all β-mannosidosis-causing mutations abrogate the β-mannosidase activity. The present study indicates that this is not the case. The c.1922G>A (p.Arg641His) mutation was associated with a residual β-mannosidase activity of about 7% in the patient's leukocytes, 11% in lymphoblasts and 14% in plasma. These residual activities appear substantially higher than those found in cells derived from other β-mannosidosis patients, which usually ranged between 0 and 4% of the control (additional file 1; see also [23]). Moreover, the enzyme activity measured for this mutant β-mannosidase expressed in eukaryotic cells represented 7% of the wt, which is higher than that reported when three different mutant enzymes (p.Arg182Trp, p.Gly392Glu and p.Ser505Pro) were expressed in mammalian cells [24]. Thus, although markedly reduced, the β-mannosidase activity of the p.Arg641His mutant was not totally abolished. The c.1922G>A mutation leads to the replacement of an arginine by a histidine (p.Arg641His). Arginine in this position is conserved in most mammals, zebrafish, fruitfly, mosquito and worm, but is replaced by a serine in chimpanzee, by glutamine in opossum and by methionine in frog. Arginine and histidine share basic properties, and this change may allow for some residual activity. Further studies are needed to understand more about how this substitution affects the MANBA protein.

Despite its residual activity, the p.Arg641His mutant enzyme is associated with a severe clinical presentation ([22]; see also additional file 1). It has been proposed that the nature of the *MANBA *mutation dictates the severity of the β-mannosidosis phenotype. Null mutations could have been responsible for the most severe phenotypes while missense mutations could have led to the mildest ones. However, recent findings have challenged this view, by showing that null mutations are sometimes associated with mild phenotypes [23]. On the other hand, some missense mutations completely abrogate the enzyme activity, and thus could not explain mild phenotypes [24]. The present study further extends this lack of genotype-phenotype correlation by showing that a mutation that does not fully abolish the enzyme activity is associated with a severe form of the disease (the possibility that the observed residual enzyme activity may exist only towards the artificial fluorogenic substrate, but not the natural substrate, cannot be excluded). We suggest that the level of enzymatic activity is not sufficient to predict the clinical severity, and that, as previously concluded [23,24] other, epigenetic or environmental factors contribute to the clinical heterogeneity of β-mannosidosis. Alternatively, the p.Arg641His mutant may represent the first report of β-mannosidase pseudodeficiency. Although it has never been reported so far for this particular enzyme, pseudodeficiencies of other lysosomal hydrolases are well known [26]. Enzyme residual activities of previously reported pseudodeficiency cases can be quite variable. It is therefore difficult to use the degree of residual activity as a sole means to differentiate between a pseudodeficiency and a disease-causing defect. In the present case, the hypothesis of a pseudodeficiency would be in accordance with the fact that no oligosacchariduria was detected [22]. If so, the patient's symptomatology would not be the result of the observed reduced enzyme activity but a yet unkown cause.

## Conclusion

The present analysis of the c.1922G>A *MANBA *mutation underlines the lack of genotype-phenotype correlation in beta-mannosidosis. The hypothesis that this mutation leads to a pseudodeficiency cannot yet be ruled out.

## Abbreviations

HEK: human embryonic kidney cells; wt: wild-type.

## Competing interests

The authors declare that they have no competing interests.

## Authors' contributions

FS carried out the mutagenesis studies, the enzymatic analysis and drafted the manuscript. PL DR, GC and NdC performed the clinical evaluation of the patient. MN, VL and HMFRS carried out the molecular genetic studies. TL conceived of the study, participated in its design and coordination, and prepared the final version of the manuscript. All authors read and approved the final manuscript.

## Pre-publication history

The pre-publication history for this paper can be accessed here:



## Supplementary Material

Additional file 1 **Table 2**. Residual enzyme activity of β-mannosidase and clinical phenotype associated by *MANBA *mutations. Click here for file

## References

[B1] Jones MZ, Dawson G (1981). Caprine beta-mannosidosis. Inherited deficiency of beta-D-mannosidase. J Biol Chem.

[B2] Wenger DA, Sujansky E, Fennessey PV, Thompson JN (1986). Human beta-mannosidase deficiency. N Engl J Med.

[B3] Cantz M, Ulrich-Bott B (1990). Disorders of glycoprotein degradation. J Inherit Metab Dis.

[B4] Cherian MP (2004). Beta-mannosidae deficiency in two mentally retarded girls with intractable seizures. Ann Saudi Med.

[B5] Cooper A, Hatton C, Thornley M, Sardharwalla IB (1988). Human beta-mannosidase deficiency: biochemical findings in plasma, fibroblasts, white cells and urine. J Inherit Metab Dis.

[B6] Cooper A, Sardharwalla IB, Roberts MM (1986). Human beta-mannosidase deficiency. N Engl J Med.

[B7] Cooper A, Wraith JE, Savage WJ, Thornley M, Noronha MJ (1991). beta-mannosidase deficiency in a female infant with epileptic encephalopathy. J Inherit Metab Dis.

[B8] Dorland L, Duran M, Hoefnagels FE, Breg JN, Fabery de Jonge H, Cransberg K, van Sprang FJ, van Diggelen OP (1988). Beta-mannosidosis in two brothers with hearing loss. J Inherit Metab Dis.

[B9] Gort L, Duque J, Fabeiro JM, Zulaica A, Coll MJ, Chabas A (2006). Molecular analysis in two beta-mannosidosis patients: description of a new adult case. Mol Genet Metab.

[B10] Gourrier E, Thomas MP, Munnich A, Poenaru L, Asensi D, Jan D, Leraillez J (1997). [Beta mannosidosis: a new case]. Arch Pediatr.

[B11] Kleijer WJ, Hu P, Thoomes R, Boer M, Huijmans JG, Blom W, Van Diggelen OP, Seemanova E, Macek M (1990). Beta-mannosidase deficiency: heterogeneous manifestation in the first female patient and her brother. J Inherit Metab Dis.

[B12] Levade T, Graber D, Flurin V, Delisle MB, Pieraggi MT, Testut MF, Carriere JP, Salvayre R (1994). Human beta-mannosidase deficiency associated with peripheral neuropathy. Ann Neurol.

[B13] Molho-Pessach V, Bargal R, Abramowitz Y, Doviner V, Ingber A, Raas-Rothschild A, Ne'eman Z, Zeigler M, Zlotogorski A (2007). Angiokeratoma corporis diffusum in human beta-mannosidosis: Report of a new case and a novel mutation. J Am Acad Dermatol.

[B14] Poenaru L, Akli S, Rocchiccioli F, Eydoux P, Zamet P (1992). Human beta-mannosidosis: a 3-year-old boy with speech impairment and emotional instability. Clin Genet.

[B15] Rodriguez-Serna M, Botella-Estrada R, Chabas A, Coll MJ, Oliver V, Febrer MI, Aliaga A (1996). Angiokeratoma corporis diffusum associated with beta-mannosidase deficiency. Arch Dermatol.

[B16] Sedel F, Friderici K, Nummy K, Caillaud C, Chabli A, Durr A, Lubetzki C, Agid Y (2006). Atypical Gilles de la Tourette Syndrome with beta-mannosidase deficiency. Arch Neurol.

[B17] Suzuki N, Konohana I, Fukushige T, Kanzaki T (2004). Beta-mannosidosis with angiokeratoma corporis diffusum. J Dermatol.

[B18] Uchino Y, Fukushige T, Yotsumoto S, Hashiguchi T, Taguchi H, Suzuki N, Konohana I, Kanzaki T (2003). Morphological and biochemical studies of human beta-mannosidosis: identification of a novel beta-mannosidase gene mutation. Br J Dermatol.

[B19] Wijburg H, de Jong J, Wevers R, Bakkeren J, Trijbels F, Sengers R (1992). Beta-mannosidosis and ethanolaminuria in a female patient. Eur J Pediatr.

[B20] Abbitt B, Jones MZ, Kasari TR, Storts RW, Templeton JW, Holland PS, Castenson PE (1991). Beta-mannosidosis in twelve Salers calves. J Am Vet Med Assoc.

[B21] Jones MZ, Abbitt B (1993). Animal model of human disease. Bovine beta-mannosidosis. Am J Pathol.

[B22] Labauge P, Renard D, Castelnovo G, Sabourdy F, de Champfleur N, Levade T (2009). Beta-mannosidosis: a new cause of spinocerebellar ataxia. Clin Neurol Neurosurg.

[B23] Bedilu R, Nummy KA, Cooper A, Wevers R, Smeitink J, Kleijer WJ, Friderici KH (2002). Variable clinical presentation of lysosomal beta-mannosidosis in patients with null mutations. Mol Genet Metab.

[B24] Riise Stensland HM, Persichetti E, Sorriso C, Hansen GM, Bibi L, Paciotti S, Balducci C, Beccari T (2008). Identification of two novel beta-mannosidosis-associated sequence variants: biochemical analysis of beta-mannosidase (MANBA) missense mutations. Mol Genet Metab.

[B25] Alkhayat AH, Kraemer SA, Leipprandt JR, Macek M, Kleijer WJ, Friderici KH (1998). Human beta-mannosidase cDNA characterization and first identification of a mutation associated with human beta-mannosidosis. Hum Mol Genet.

[B26] Thomas GH (1994). "Pseudodeficiencies" of lysosomal hydrolases. Am J Hum Genet.

