# Supplementary material for: A MANBA mutation resulting in residual beta-mannosidase activity associated with severe leukoencephalopathy: a possible pseudodeficiency variant
Source: BMC Med Genet. 2009 Sep 3;10:84. doi: 10.1186/1471-2350-10-84 (PMC2745377; doi:10.1186/1471-2350-10-84)
Supplement: Additional file 1 — Table 2. Residual enzyme activity of β-mannosidase and clinical phenotype associated by MANBA mutations. [file 1471-2350-10-84-S1.pdf]

**Table 2. Residual enzyme activity of β-mannosidase and clinical phenotype associated by MANBA mutations.**

| Mutation | | | Activity in patients' cells (patient vs. normal) * | Activity in transfected COS-7 cells | Symptoms | Ref. for molecular analysis | Ref. for clinical analysis |
|---|---|---|---|---|---|---|---|
| Position | DNA | Protein | | | | | |
| Exon 2 | c.248G>T | p.Glu83X | Leukocytes : 1 vs. 245-467 Fibroblasts : 4 vs. 58-389 | ND | Age of onset : 5 years. Hearing loss, mental retardation. No other neurological signs. Angiokeratomas. No facial dysmorphism, no skeletal deformation. | [23] | [19] |
| Exon 10 | c.1276C>T | p.Gln426X | | | | | |
| Exon 3 | c.375A>G | p. Tyr126AlafsX11 | Leukocytes: 0.5 vs. 104-432 Fibroblasts: 0 vs. 60-208 | ND | Infantile onset. Speech impairment, mental retardation, hyperactive behavior, facial dysmorphism and respiratory infections. No skeletal deformations. | [24] | [9] |
| Exon 12 | c.1513T>C | p.Ser505Pro | | 0 | | | |
| Exon 4 | c.544C>T | p.Arg182Trp | Fibroblasts : 1.5% Serum and leukocytes : not detectable | 0 | Age of onset : 24 years. Angiokeratoma, slight deafnesss, abdominal pain. No neurological involvement. | [17, 24] | [17] |
| Exon 11 | c.1398G>A | p.Trp466X | | ND | | | |
| Exon 5 | c. 563_572dup10 | p.Trp192X | Leukocytes: 18 vs. 469 U/L Serum: 24 vs. 407 U/L | ND | Age of onset : 18 months. Delay in speech acquisition. Motor and vocal tics, attention-deficit/hyperactivity disorder, aggressiveness compatible with Gilles de la Tourette syndrome. Recurrent upper airway infections, | [16] | [16] |
| Intron 13 | c.1705-1G>A | not applicable | | ND | | | |

| | | | | | | | |
|---|---|---|---|---|---|---|---|
| | | | | | hearing loss. No other neurological problems. No dysmorphism. | | |
| Exon 6 | c.693G>A | p.Trp231X | Leukocytes, fibroblasts and serum :<1% | ND | Mental retardation since childhood, aggressive behavior, deafness, recurrent erysipelas, mild renal failure. Multiple angiokeratomas. | [18] | [18] |
| Exon 9<br>Exon 13 | c.1175G>A<br>c.1848delA | p.Gly392Glu<br>p.Asp617IlefsX8 | Leukocytes : 3 vs. 73-186 Fibroblasts : 1.9 and 5 vs. 98-428 | 0<br>ND | Age of onset : 12 years. Angiokeratoma, scantly communicative. No mental retardation, no hearing loss, no behavior problems, no neurological skeletal deformation, no respiratory signs, no facial dysmorphism, no infections. | [17, 24] | [11] |
| Exon 11 | c.1454-1455delAT | p. Tyr485CysfsX27 | Leukocytes : 4.7 vs. 160-300 Fibroblasts : 0 vs. 115-455 | ND | Age of onset : 15 months. Speech development problems. Recurrent respiratory tract infections, hearing loss. Motor and mental retardation (at age 5), hyperactive and aggressive behavior (at age 18), facial dysmorphism and skeletal deformation. | [23] | [8] |
| Intron 7 | c.960+1G>A | p.Val321IlefsX5 | Plasma: 5.4 vs.193-331 Fibroblasts: 10% | ND | Age of onset : 2 years. Hearing loss, mental retardation and developmental disorders. Hypertension and chronic renal failure. Numerous angiokeratomas. | [13] | [13] |

| | | | | | | | |
|---|---|---|---|---|---|---|---|
| Intron 15 | c.2015-2A>G | p.Val720LeufsX6<br>p.Val720AlafsX75 | Fibroblasts :<br>0 vs. 51-195 | ND | Age of onset : 14 months. Severe psychomotor retardation, bone deformities and facial dysmorphism, macroglossia, gingivial hyperplasia, umbilical hernia. Recurrent skin and respiratory infections. Hypotonia, aggressive behavior. | [25] | [6] |
| | | | Leukocytes :0<br>Fibroblasts : 0<br>Plasma : 0 | | Affected brother: milder facial dysmorphism, mental retardation, hearing impairment and recurrent infections. | | [6] |
| ND | ND | ND | Leukocytes:<br>0 vs. 119<br>Fibroblasts:<br>0 vs. 103 | ND | Age of onset : 1 year. Facial dysmorphism and skeletal deformations, mental retardation, hyperactive behavior, mild hearing loss. (+ sulfamidase deficiency) | | [2] |
| ND | ND | ND | Leukocytes:<br>0 vs. 45-150 | ND | Age of onset : 3 weeks. Mental retardation, hearing loss, hypotonia, speech retardation, feeding difficulties, respiratory infections. No facial dysmorphism, no skeletal deformation. | | [5] |
| ND | ND | ND | Leukocytes:<br>7 vs. 245-467<br>Fibroblasts:<br>8.7 vs. 58-389 | ND | Age of onset : 9 months. Seizures, moderate developmental delay. Brachecephaly. No skeletal deformation. Death at 15 months. | | [7] |

| | | | | | | |
|---|---|---|---|---|---|---|
| ND | ND | ND | Leukocytes: 3 vs. 80-170 | ND | Age of onset : 6 years. Disinterest, apathy, clumsiness, demyelinating peripheral neuropathy. No facial dysmorphism, no skeletal deformation, no hearing loss. | [10] |
| ND | ND | ND | Serum : 22 vs. 180-600 Leukocytes: 0 vs. 100-450 | ND | Age of onset : 7 months. Hypotonia, feeding difficulties, recurring respiratory infections, delayed motor development. | [12] |

* Unless otherwise indicated, enzyme activity is expressed as nmol/h.mg protein for leukocytes or fibroblasts, or nmol/h.ml for serum. ND, not determined.
